# Towards improving sterile insect technique: Exposure to orange oil compounds increases sexual signalling and longevity in *Ceratitis capitata* males of the Vienna 8 GSS

**DOI:** 10.1371/journal.pone.0188092

**Published:** 2017-11-30

**Authors:** Nikos A. Kouloussis, Christos D. Gerofotis, Charalampos S. Ioannou, Ioannis V. Iliadis, Nikos T. Papadopoulos, Dimitris S. Koveos

**Affiliations:** 1 Laboratory of Applied Zoology and Parasitology, School of Agriculture, Aristotle University of Thessaloniki, Thessaloniki, Greece; 2 Department of Agriculture Crop Production and Rural Environment, University of Thessaly, Ionia (Volos), Greece; University of Crete, GREECE

## Abstract

The Mediterranean fruit fly (medfly), *Ceratitis capitata*, is a notorious insect pest causing huge economic losses worldwide. The sterile insect technique (SIT) is widely used for its control. Using sexually mature sterilized males of the Vienna 8 (tsl) strain in the laboratory, we explored whether exposure of males to citrus compounds (separately or in a mixture) affects their sexual behaviour and if nutritional conditions and age modulate those effects. Exposed males exhibited increased sexual signalling compared to unexposed ones, particularly when fed a rich adult diet. Interestingly, and for the first time reported in medfly, exposure of Vienna 8 males to a mixture of citrus compounds increases longevity under poor adult diet conditions. We discuss the possible associated mechanisms and provide some practical implications of our results towards improving the effectiveness of SIT.

## Introduction

The sterile insect technique (SIT) is an environmentally friendly approach for suppressing or eradicating insect pests of medical and agricultural importance. During SIT operations, overwhelming numbers of mass reared, sterilized male insects are released systematically in the field to compete against feral males for wild females. Females that copulate with sterilized males lay infertile eggs and therefore, the target population is suppressed [1]. The success of SIT depends on a variety of abiotic (environmental) and biotic factors that affect the quality of produced insects (i.e. sterile males’ dispersal ability, survival and mating competitiveness in the wild) [2].

The Mediterranean fruit fly (medfly) *Ceratitis capitata* (Diptera: Tephritidae) has long tradition as a model organism in SIT applications and studies. The medfly is one of the most destructive agricultural insect pests worldwide, capable of infesting more than 300 species of fruits, nuts and vegetables causing extensive economic damages [3, 4]. Nowadays all SIT programs against *C*. *capitata* include male only releases and this was achieved by the development of genetic sexing strains (GSS) [5]. Genetic sexing strains offer easy and secure separation of males from females during the rearing processes, using the pupal colour and the presence of temperature-sensitive lethal (*tsl*) genes as selectable markers [5, 6]. Among the different GSS that have been developed for the Mediterranean fruit fly, the Vienna 8 (*tsl*) strain is currently used in most mass rearing facilities and SIT programs worldwide.

During the establishment and mass rearing processes, medflies are subjected to both strong genetic selection and overcrowded conditions that induce adverse effects on males’ behavioral traits. These include reduced lifespan, low ability to escape from predators, low adaptability to environmental conditions and changes in daily sexual activity relative to their wild counterparts/competitors (reviewed by [7]). Moreover, in several cases the use of γ-irradiation to sterilize insects has been found to further reduce medfly males’ overall mating performance and quality [8]. However, in other studies no major effects of irradiation have been depicted [9].

Plant chemicals appear to have a strong influence on the mating behavior of tephritid fruit flies, including *C*. *capitata*. Medfly males are strongly attracted to the odors of both citrus and ginger (*Zingiber officinale* Roscoe) root essential oils, while exposure (by contact or vapour) to these plant chemicals boosts their mating success considerably [10–15]. The underlying mechanism of such an increase in mating success seems to be linked to higher rates of sexual signaling (i.e. release of sex pheromone from the abdomen [16]) of exposed males relative to non-exposed ones in such compounds [17]. Studies document a direct, positive relationship between “signalling” propensity and mating success in male medflies [18, 19] and propose the hydrocarbon sesquiterpene *α-*copaene- a powerful attractant to male medflies present in both citrus and ginger root oil- to be responsible for this phenomenon [13, 14, 20]. However, recent work demonstrates that citrus compounds, other than *α-*copaene, may also confer a significant mating advantage on *C*. *capitata* males. As such, males’ exposure to the odors of the oxygenated monoterpene linalool, as well as to a mixture consisted of equal amounts of pure linalool, limonene, *α-*pinene, *β*-myrcene and geraniol, significantly improved their mating competitiveness [21,22].

The mating performance of sterilized medfly males is influenced by the pre-release nutritional conditions [23] and the age of individuals [24–28]. Carbohydrates are essential for energy demanding activities such as flight and dispersal, while protein consumption (same with yeast hydrolyzate) is tightly associated with reproduction [29, 30]. Protein addition to the adult diet enhances male sexual signalling activity and copulatory success relative to males fed only to sugars [31–35]. However, for mass reared males, these effects differentiate on the lab strain, experimental and environmental conditions (see in [2] and references therein). Moreover, protein consumption has been shown to affect other biological characteristics in males, equally important to SIT. For example, protein-restricted adults survive for a shorter period compared to those feeding on protein [23]. SIT programs rely on the survival of the released males, so as to minimize the need of continuous releases (and thus lead to a more cost effective operations) of millions of flies produced aiming to induce sterility in wild female populations [1]. On the other hand, age affects the mating performance of sterilized male medflies. Sterilized males can mate with females from the age of 3 days (and thus start mating related activities like lek aggregation, signalling and courtship behaviour) [36]. Male ageing may affect their courtship ability and/or pheromone production and longer sexual calling was found in young (wild and sterilized) males compared to older ones [24, 25].

Based on our previous research and working towards improving male performance and ultimately SIT- through testing new plants compounds evoking multifaceted effects to sterilized males- and considering that such effects are context-dependent, the aims of this study were to: a) test for possible beneficial effects of citrus oil and other compounds (separately or mixed) to male sexual performance on Vienna 8 genetic sexing strain, b) investigate whether such effects are modulated by the dietary conditions that adults experience and the age of the individuals and c) search for possible effects of these substances other than enhancement of male sexual behaviour, that might be useful to SIT experts.

## Materials and methods

### Laboratory conditions and insects used

The experiments were conducted in the laboratory at 25±2°C, 65±5% RH, and a photoperiod of L14:D10, with photophase starting at 07:00. We used mass-reared sterilized *Ceratitis capitata* males of the VIENN*A-*8 *tsl* genetic sexing strain, which is the strain that is widely used in SIT. The flies were derived from the mother colony of this strain maintained at the Entomology Unit of the FAO/IAEA Agriculture and Biotechnology Laboratory, Seibersdorf, Austria. Following emergence, 200 sterilized VIENN*A-*8 *tsl* males were placed in well-ventilated wooden 30 x 30 x 30 cm cages. Each cage contained water and as food either only sugar (S) or a protein mixture (PS) of yeast hydrolysate (ICN Biomedicals Inc.) and sugar in a ratio of 1:4. It should be clarified that protein is the same as yeast hydrolyzate and refer to the same adult diet that is widely used in Tephritid fruit flies. Hereafter, we will use the term yeast hydrolyzate (YS).

### Exposure of males to citrus oil, limonene and the mixture

The experiment involved exposure of males to orange oil, limonene, or the mixture of five orange oil compounds that is known to enhance male mating competitiveness. The mixture consisted of equal quantities (1:1:1:1:1) of linalool, geraniol, *α*-pinene, limonene and *β-*myrcene. Precise information on the purity, components technical details and on the preparation of the mixture are provided by [22]. In each cage, we placed a 5-cm-diam dome polyethylene hemisphere fitted to a similarly-size hole opened in the middle of a 5.5 cm petri dish lid. The dome was punctured with 100 evenly spaced 1-mm-diam holes. For the exposure, we applied 25 μl of the treatment substance (or water in the case of the control) with a micro capillary pipette to a 3-cm-diam piece of white filter paper placed in the center of the Petri dish base (S6 Fig). Volatiles from the three treatments (or water vapors from the control) filled the space under the dome and emanated outside it through the holes. Exposure was done on day 4 and repeated with new compounds on day 5. During exposure days, control males were kept in a different room to avoid inadvertent exposure to the treatment substances. Following exposure, males were introduced into Plexiglas cages (15 X 15 X 15 cm) that had a round (ca. 10 cm diameter) nylon-mesh-screened window in one side for ventilation. Each cage contained water and the respective food (mixture of yeast hydrolyzate and sugar or sugar only). Ten such cages were used per treatment, each containing 10 males. The few males (<5%) that died during the experiment were replaced with ones of the same age and treatment. Sexual signalling was recorded at hourly intervals from 7:00 to 20:45 (last observation was conducted 15 min earlier to facilitate observation before lights turned off). Preliminary observation showed that sexual signalling in this strain started after day 5 and lasted until at least day 16 of their adult life. Observations were thus conducted during days 6–10 and on day 16. Each cage was observed for a few seconds and the number of males signalling at the time was recorded. Signalling males were distinguished easily from non-signalling ones, since they keep their abdomen curled upward with a bubble-like structure appearing at the end [16].

### Daily pattern of signalling activity

The daily pattern of sexual signalling activity for each exposure compound and food regime was determined by analyzing the hourly records (07:00–20:45 hours) from day 6–10 and once more at day 16. Original data are given in a separate excel file in Supplementary material (S1 File)

### Effect of citrus oil to male longevity

Soon after sexing males were placed in groups of 200 in wooden cages (30X 30 X 30 cm bearing two 225 cm^2^ windows covered with mesh cloth for ventilation) with water and adult food (ad libitum) consisting of either a mixture of yeast hydrolyzate (mentioned also as protein) (MP Biomedicals LLC, France), sugar and water at 1:4:5 or a mixture of sugar and water. On day 3 and 4 of adult life, half of males were exposed to 25μl of citrus oil (Sigma Aldrich, Karlsruhe) and half to water (control). Therefore, we established four male cohorts. Males fed in full diet conditions (access to the mixture of yeast hydrolyzate and sugar) that were either (a) exposed or (b) non-exposed (control) to citrus oil. Similarly, in poor food conditions (access only to sugar) males were either exposed (c) or non-exposed (control) to citrus oil (d). On day 5 (one day after last exposure to citrus oil), males of each group were transferred and held individually till the end of adult life into transparent plastic cages (400-ml capacity plastic cups) bearing a 24 cm^2^ lateral opening covered with mesh cloth to allow adequate ventilation and offered the respective adult food they had experienced before. Male lifespan was recorded daily until the death of the last individual in the cohort. One hundred individuals were considered for each treatment. Original data are given in a separate excel file in Supplementary material (S2 File).

### Statistical analysis

The effect of exposure compound (factor), food (factor), and age (days as a repeated factor) on male sexual signalling behaviour was determined by repeated measures analysis of variance (ANOVA) [37]. The effect of exposure compound (factor), food (factor) and time of the day (repeated factor) on the daily pattern of male sexual signalling was also determined by repeated measures ANOVA performed separately for each of the adult days 6–10 and also day 16. As the results obtained from those analyses were similar we present data only for adult day 6 (the rest of them are presented in S1–S5 Figs). To quantify the effect of YS on the signalling levels for each exposure treatment, we calculated the average daily signalling ratio between YS- and S-fed males in relation to the age of males. These data were analyzed by a multiple linear regression analysis (ANCOVA) with ratio (YS/S) as a dependent variable, and age after exposure day (6–16 days) and exposure compound as independent variables. Comparisons of means were performed using Tukey’s HSD test. In cases where data were not normally distributed despite all possible transformations, the analysis was performed using the nonparametric Kruskal–Wallis *H*-test; means were separated by the Mann–Whitney *U*-test. Pairwise comparisons were performed using the independent *t*-test. Male longevity was analyzed using the Kaplan-Meier rates to estimate the survival function followed by the log-rank test to compare the survival distributions [38]. Pairwise log-rank tests were adjusted for comparisons between exposed and non-exposed males to orange oil in the two food regimes. All analyses were conducted using SPSS, version 21 (SPSS, IBM).

## Results

### Effect of exposure compound, age and food regime

**Fig 1:**
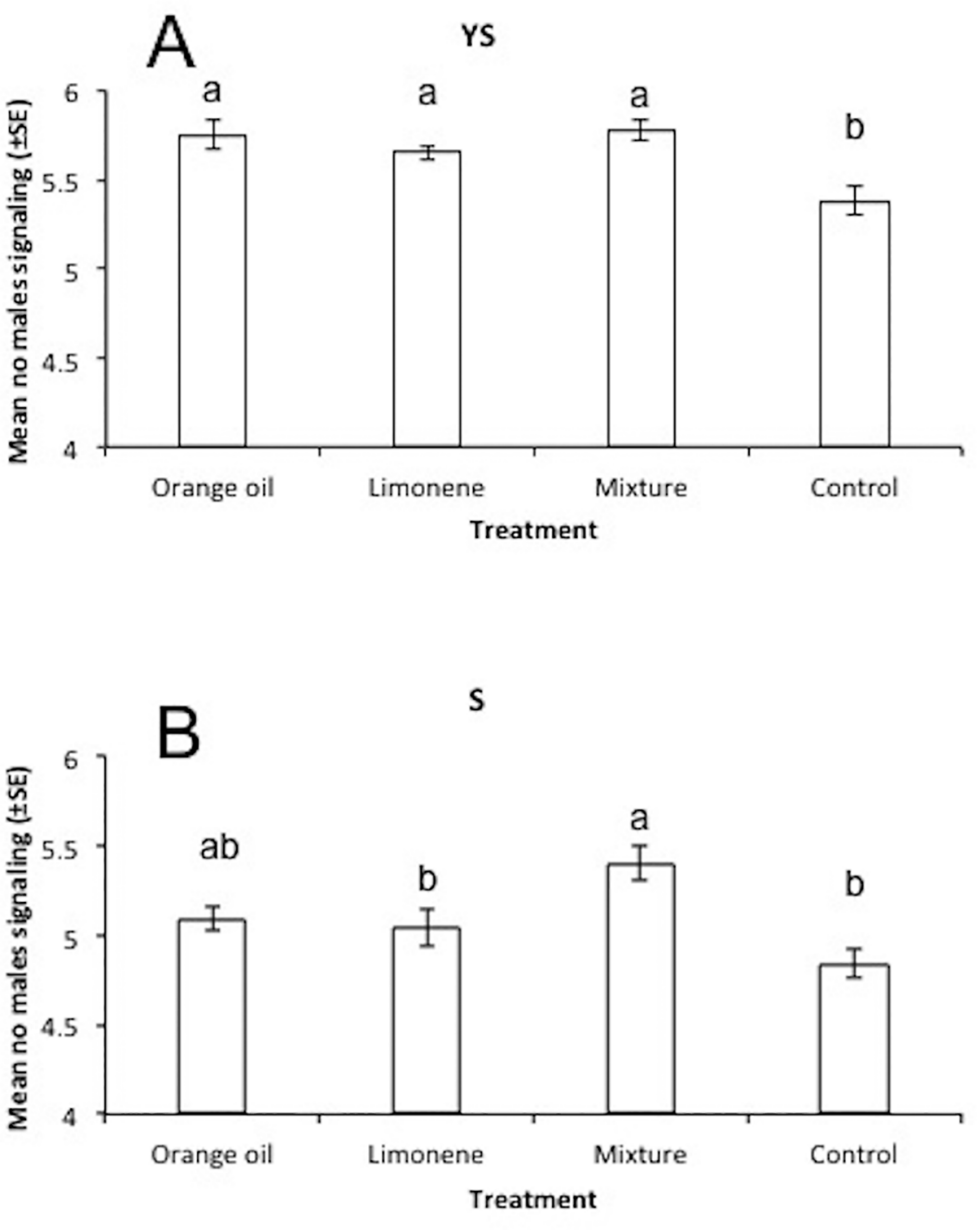
Effects of exposure to citrus compounds on male sexual signaling. Overall levels of sexual signalling activity (mean number of males signalling through the ages of 6 to 10 days old and 16 days old) of Vienna 8 GSS sterilized males medflies that were exposed during day 4 and 5 of adult life to orange essential oil, limonene, and a mixture of 5 pure compounds (limonene, linalool, myrcene, *α*-pinene and *β*-myrcene 1:1:1:1:1 ratio) or left unexposed (control) fed on (A) yeast hydrolyzate & sugar (YS) and (B) sugar only (S). On each day of age, observations took place hourly from 07:00 to 20:45 hours in 10 cages (replicates) containing 10 males each. Values on y-axis are mean numbers (±SE) of males signalling per cage per hour observation. Means followed by the same lowercase letter are not significantly different (P>0.05, Tukey’s HSD test).

**Fig 2:**
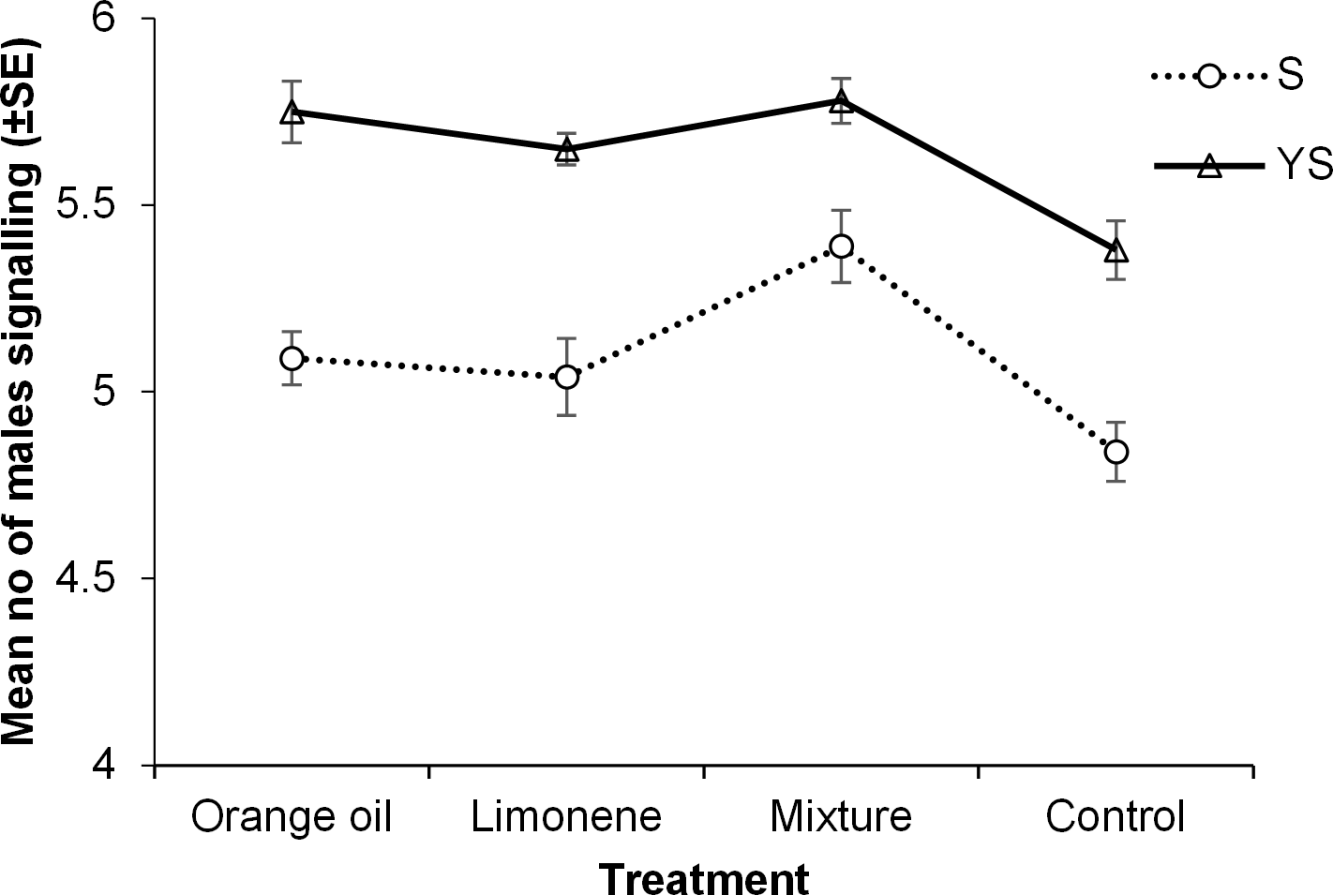
Diet specific effects on male sexual signaling. Effect of yeast hydrolyzate and sugar (YS) and sugar only (S) on the overall levels of sexual signalling activity (mean daily activity through the ages of 6 to 10 days old and 16 days old) of Vienna 8 GSS sterilized male medflies, that were exposed during day 4 and 5 of adult life to orange essential oil, limonene and a mixture of 5 pure compounds (limonene, linalool, myrcene, *α*-pinene and *β*-myrcene, 1:1:1:1:1 ratio) or left unexposed (control). On each day of age, observations took place hourly from 07:00 to 20:45 hours in 10 cages (replicates) containing 10 males each. Values on y-axis are mean numbers (±SE) of males signalling per cage per hour observation (** P<0.001, *P<0.01, *t*-test YS vs S in each treatment).

**Fig 3:**
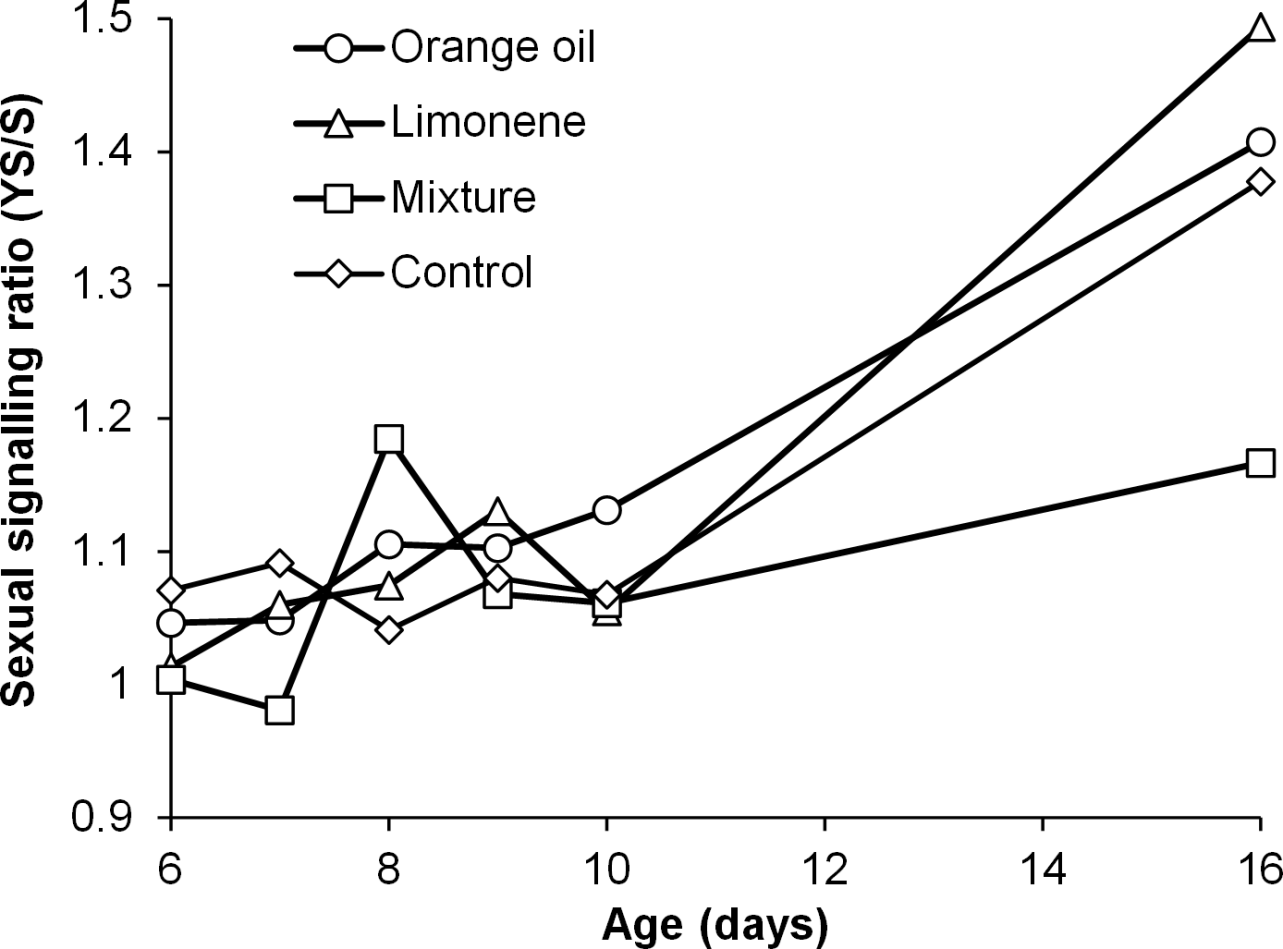
Age specific ratio on male sexual signaling. Ratio between signalling rates of Vienna 8 GSS sterilized male medflies fed on either yeast hydrolyzate & sugar (YS) or sugar only (S) in relation to age. Males had been exposed during day 4 and 5 of adult life to orange essential oil, limonene, and a mixture of 5 pure compounds (limonene, linalool, myrcene, *α*-pinene and *β*-myrcene, 1:1:1:1:1 ratio) or left unexposed (control). On each day of age, observations took place hourly from 07:00 to 20:45 hours in 10 cages (replicates) containing 10 males each. The ratio was estimated considering the average calling for each day of the observation during 07:00 to 20:45 for protein and sugar treatments.

Both exposure compound and food regime had significant effects on the overall (mean daily activity through the whole experimental period) levels of males’ sexual signalling activity (Table 1). However, no significant interaction between these two factors was observed, suggesting probably an independent mode of function on this behavioral parameter (Table 1). Males exposed to limonene, orange oil and the mixture performed significantly higher overall signalling activity relative to controls (unexposed) when they had access to YS (yeast hydrolyzate and sugar) while only the mixture had a strong impact in the case of sugar diet only (Tukey’s HSD test P<0.05) (Fig 1). Yeast hydrolyzate significantly increased sexual signalling in all four treatments relative to sugar only (t-test, P<0.05) (Fig 2). The positive effect of YS on the signalling levels of each treatment in relation to the age of sterilized males is given in Fig 3. While at younger ages (6 to 10 days) YS availability had a weak effect on signalling activity, at day 16 it boosted signalling levels approximately 1.4 times in all cases except the mixture. Multiple regression analysis revealed that treatment was not a significant predictor of the YS/S ratio of sexual signalling (F_3,216_ = 1.7, P = 0.167), while age was (F_5,216_ = 17.7, P< 0.001). The positive effect of YS on sexual signalling was significantly higher on day 16 of males’ age relative to others (Mann-Whitney *U*-test, P<0.05).

**Table 1:** Repeated measures ANOVA on the effect of exposure compound (orange oil, limonene, mixture of 5 pure compounds and control), food (yeast hydrolyzate and sugar and sugar only) and age (repeated factor) on sexual signalling on sterilized (Vienna 8 GSS), *Ceratitis capitata* males. Males from adult age day 6 to adult day 10 and adult day 16 were considered in the analysis.

Source of variation	d.f.	MS	F	P
Exposure compound	3	4.63	12.43	<0.001
Food	1	36.48	97.95	<0.001
Exposure compound*food	3	0.40	1.08	0.365
Error (between subjects)	72	0.37	-	-
Age	5	4.87	13.51	<0.001
Age*exposure compound	15	0.28	0.77	0.714
Age*food	5	4.49	12.45	<0.001
Age*exposure compound*food	15	0.40	1.10	0.358
Error (age)	360	0.36	-	-

### Daily rhythm of sexual signalling on Vienna 8 GSS sterilized males

**Fig 4:**
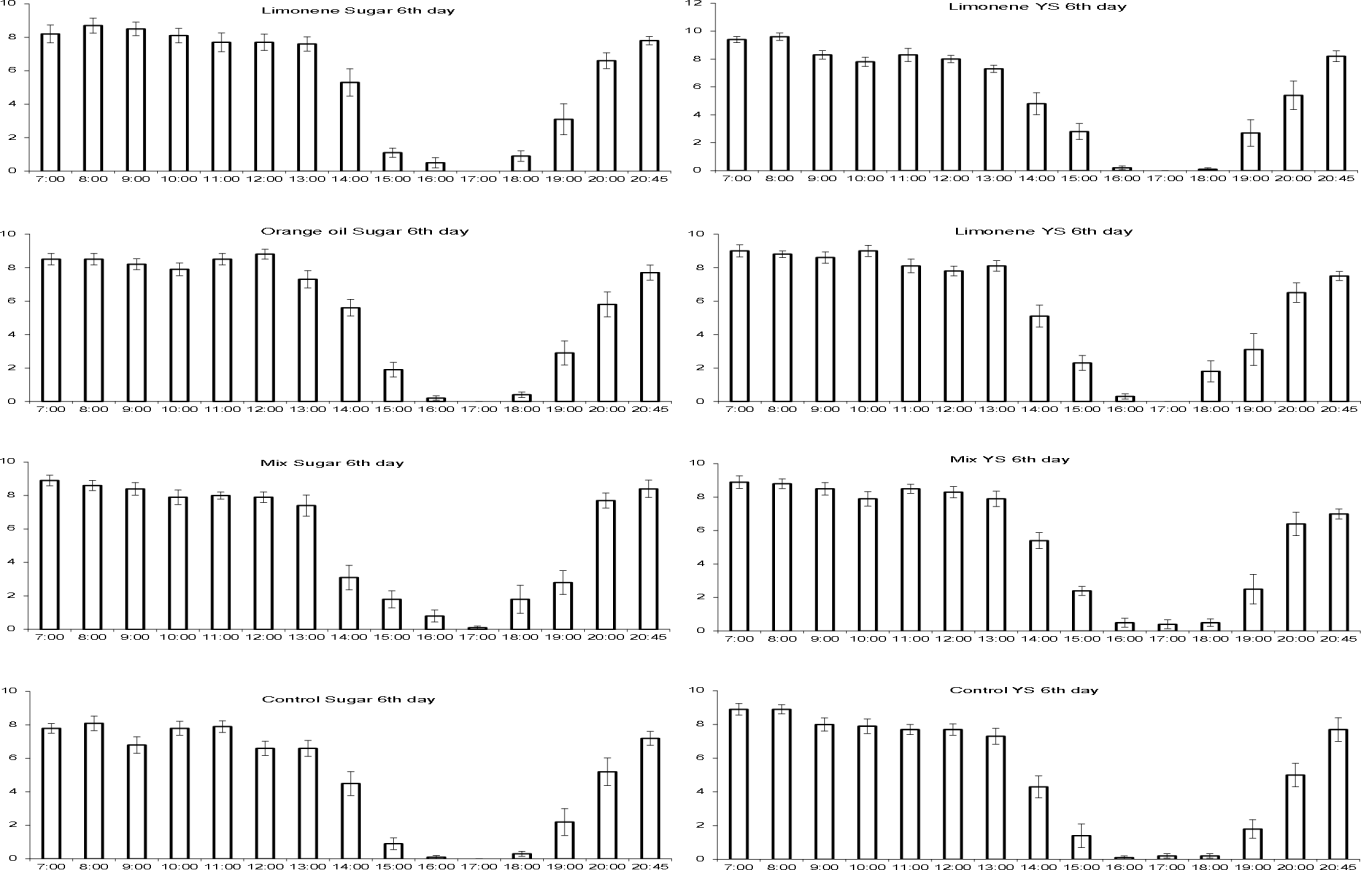
Daily pattern on male sexual siganlling. Daily rhythm of sexual signalling on adult day 6 of sterilized male *C*. *capitata* of the Vienna 8 GSS in four different exposure compounds (orange oil, limonene, mixture of 5 pure compounds and control) on yeast hydrolyzate & sugar (YS, left column) and sugar only (S, right column). Values on y axis indicate the mean number (±SE) of males signalling per cage. Ten cages were considered containing 10 males each.

Considering counts at day 6 of age (one day soon after males’ exposure to citrus chemicals), exposure compound but not food significantly affected sexual signalling pattern. Time of the day had a strong impact on the patterns of signalling activity (Table 2, Fig 4). Regardless of exposure compound and food regime, sexual signalling activity followed a bimodal pattern with one clear peak and plateau between 7:00 and 13:00 hours and the second at the end of the photophase (20:45) (Fig 4). On the other hand, both exposure compound and food had a significant influence on sexual signalling pattern at day 8 of age (three days after males’ exposure to citrus chemicals) (S1 Table). The exposure compound*food interaction was also significant. Again, time of the day had a strong influence on the signalling rates, which followed a similar pattern to day 6 of males’ age (S1 Fig). The interaction between time of day and exposure compound was significant at day 8 of males’ age, suggesting different daily patterns of signalling among the different treatments. Food regime in turn may also have caused shifts in the daily patterns of signalling rates as indicated by the significant time of day*food interaction (S1 Table). In general, these differences in sexual signalling activity mostly occurred during the “relaxation” period from 14:00 to 18:00 hours (S1 Fig). Finally, considering counts at day 16 of sterile males’ age, only food but not exposure compound significantly affected sexual signalling pattern, suggesting a diminishing effect of citrus chemicals 11 days post exposure (S2 Table). The interaction between time of day and food was significant indicating different daily patterns of signalling between the two food regimes. More specific, YS-fed males exhibited higher signalling activity relative to S-fed ones during the whole observation period but mostly at the second peak at the end of the photophase (S2 Fig). Interestingly, irrespective of the food regime and exposure compound, 16 days old sterilized males were more active during the “relaxation” period (from 14:00 to 18:00 hours) relative to younger ages suggesting a shift of daily signalling rhythm with ageing (S2 Fig). Intermediate days in daily rhythm of sexual calling (i.e. days 7, 8, 9, 10 and 16) are given in the Supplementary material (S1–S5 Figs).

**Table 2:** Repeated measures ANOVA on the effect of exposure compound (orange oil, limonene, mixture of 5 pure compounds and control), food (yeast hydrolyzate and sugar and sugar only) and time of day (repeated factor) on daily rhythms of sexual signalling on sterilized (Vienna 8 GSS) *C*. *capitata* males. Times of the day between 07:00 and 20:45 hours of adult day 6 were considered in the analysis.

Source of variation	d.f.	MS	F	P
Exposure compound	3	26.87	5.79	0.001
Food	1	8.84	1.90	0.172
Exposure compound*food	3	1.69	0.36	0.779
Error (between subjects)	72	4.64	-	-
Time of the day	14	891.75	441.53	<0.001
Time of the day*exposure compound	42	1.71	0.84	0.749
Time of the day*food	14	2.71	1.34	0.176
Time of the day*exposure compound*food	42	2.67	1.32	0.084
Error (time of the day)	1008	2.02	-	-

### Effects of food and exposure compound on sterile males’ longevity

**Fig 5:**
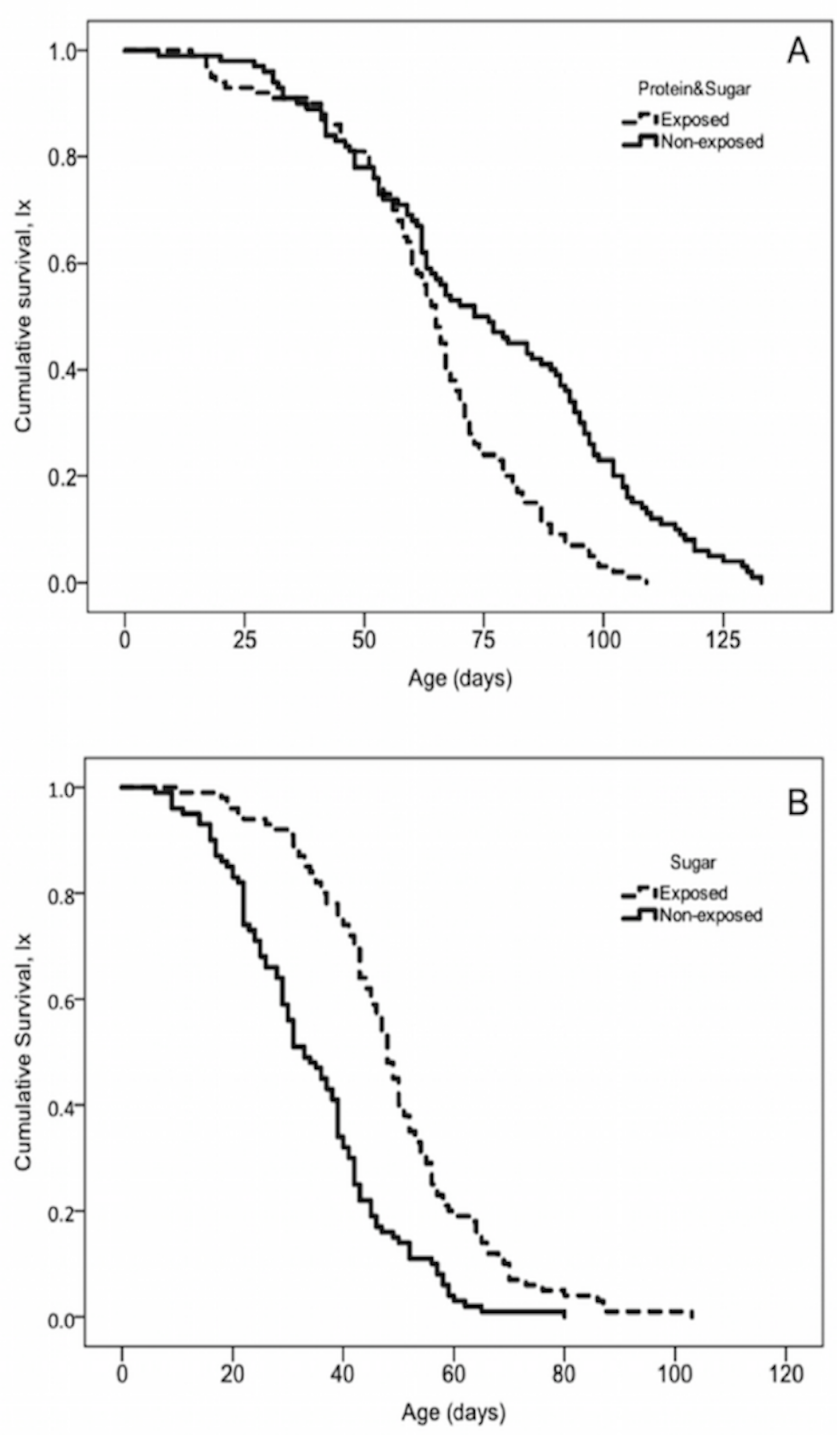
Male longevity. Age-specific survival patterns of sterilized male *C*.*capitata* of the Vienna 8 GSS that were either exposed (solid line) or non-exposed (dashed line) to citrus oil: (A) males in full diet (yeast-sugar fed) conditions, (B) male in diet restriction (sugar only fed), (Log-rank pairwise tests reveal significant differences between exposed and non-exposed males fed in sugar, *P* < 0.05).

Table 3 gives the longevity parameters of male cohorts in both food regimes and Fig 5 the respective cumulative survivorship curves. Exposure to the aroma of citrus oil significantly increased the lifespan of sugar fed males only (log-rank test; *x*^*2*^ = 37.109; df = 1; *P* < 0.001), whereas in males experienced yeast-sugar conditions lifespan was reduced (log-rank test; *x*^*2*^ = 20.677; df = 1; *P* < 0.001).

**Table 3:** Longevity parameters of adult medflies that were exposed and non-exposed to the aroma of citrus oil and held in diet restriction (sugar fed only) and in full diet (yeast and sugar fed) food conditions. Within diet regime, numbers followed by different letters are significantly different (pairwise comparisons log-rank test, P < 0.05).

Adult cohort (Number of individuals)	Longevity parameters in days ± SE			
	Average	Quartiles		
		25	50	75
**Males**				
**Yeast-sugar**				
Exposed (n = 100)	63.54 ± 2.04a	74 ± 3.46	65 ± 1.81	53 ± 2.63
Non-exposed (n = 100)	75.83 ± 2.93a	98 ± 2.99	73 ± 6.36	53 ± 4.44
**Sugar**				
Exposed (n = 100)	49.03 ± 1.59a	56 ± 1.96	48 ± 1.24	39 ± 2.59
Non-exposed (n = 100)	34.2 ± 1.44b	42 ± 1.57	33 ± 2.72	22 ± 1.25

## Discussion

Our results demonstrate that exposure to the aroma of pure citrus compounds separately and in a mixture, affect positively male (a) sexual behaviour and (b) longevity on sterilized male medflies of the Vienna 8 (tsl) genetic sexing strain. These effects (c) are modulated by the dietary context and the age of adults. Exposure of males to the tested compounds increased sexual signalling and at the same time exposure to one of them increased longevity, compared to non-exposed ones. Evoked effects (of exposure) to sexual signalling were more prominent in males that were fed in poor nutritional conditions and in older individuals. To our knowledge this is the first report on *C*. *capitata* that exposure to the odour of a plant compound (citrus oil) increases longevity and at the same time retains beneficial stimulatory effects to sexual signalling.

### Citrus compounds affect male sexual signalling and longevity

In our study, Vienna 8 sterilized males exposed to limonene, orange oil and to the mixture exhibited higher sexual signalling activity relative to unexposed ones, and to our knowledge this is demonstrated for the first time for the Vienna 8 GSS. Enhancement of sexual signalling and mating competitiveness in wild and sterile medfly males when exposed to ginger root and citrus oils has already been demonstrated in previous studies [17, 22] and these effects have been attributed to the active ingredient *α*-copaene (although clear evidence is missing). Interestingly in our study, the mixture consisting of compounds other than *α*-copaene, evoked stronger effects showing that such beneficial effects can be induced by other substances too (see also discussion in [22]). Moreover, our study highlights the necessity for future studies on investigating the role of mixing single substances, contrary to the majority of literature, which is single compound dominated.

Although the exact underlying physiological mechanisms in increasing sexual signalling in Mediterranean fruit fly males after exposure to citrus or other compounds has not been elucidated yet, it is possible that different compounds (separately or combined as a mixture) could activate alternate chemosensory pathways in males [39] (e.g. insulin signalling, neuropeptides), and thus increase sexual signalling independently, and/or each of these compounds act on the same pathway, altogether exhibiting synergistic or collective action in sexual signalling. As a response, several endocrine mechanisms could be triggered and release of hormones, like juvenile hormone (JH), could cause such effects. In the Caribbean fruit fly (*Anastrepha suspensa*) sexual signalling and production of pheromone has been linked to juvenile hormone. Topical application of JH in this species increased pheromone production significantly and newly eclosed males treated with JH exhibited higher sexual calling and at an earlier age [40]. Subsequent studies reported similar beneficial effects of JH analogues application, in increasing the sexual signalling of males in other Tephritid species (reviewed in [2]). Given that JH related effects involve evolutionary conserved signalling pathways across several species [41] we believe that further studies towards investigating the underlying physiological and molecular mechanisms should be in this direction.

In regard to the daily rhythm of the sexual calling, all males (exposed and unexposed) in our study followed a bimodal pattern and are in agreement with previous laboratory studies, in which males were held under the same laboratory conditions [31, 32]. Male medflies follow either a bimodal or a unimodal pattern and this variance has been attributed to several abiotic factors (discussed in detail by [32]). However, 3 days after exposure to the tested compounds (day 8) and within the same nutritional environments, exposed males exhibited a different pattern and were calling more intensively between the “relaxation” period (14:00 to 18:00). It seems that the physiological mechanism(s) responsible for the daily rhythm of sexual signalling require a period of time (lag phase) either to be activated by exposure to citrus compounds or alternatively the induced effects require that period to be distinct and measurable.

In addition to effects to sexual behaviour, our study provides evidence for the first time that exposure to the aroma (odour) of citrus oil modulates longevity in adult medflies. Nevertheless, modulation of life history traits- including prolongation of longevity in adult medflies- has also recently been documented by Papanastasiou and co-workers [42], but following a different experimental approach. In this study, adult medflies were in direct contact with the tested compounds (citrus oils were topically applied to the thorax of medflies), and any comparisons between the two studies are hard to make. Enhancing survival in male medflies under field conditions has also been demonstrated after exposure to ginger root oil [43], yet it is not clear if this arises from prolonged longevity per se of those males or as a side-effect of improving other traits in those males that could ultimately lead to increased longevity (e.g. greater ability to find natural sources of nutrition after release, see in [44]). Longevity regulation as a response to olfaction cues is widely documented in several model organisms [45] and recently has been reported in a Tephritid fruit fly by Gerofotis and co-workers [46], who showed that exposure of adult olive flies to the aroma of *α*-pinene modulates life-history traits. Similarly to sexual signalling, hormone and neurotransmitter variation could influence homeostatic mechanisms and evoke life-prolonging effects observed in male medflies in our study. As such, pleiotropic effects arising from JH signalling pathway could account for effects observed to behaviour and life history traits simultaneously (reviewed in [47]). Further studies on the causal mechanisms underlying exposure to the aroma of citrus compounds could provide useful information towards this direction and are now facilitated by the recent whole genome sequence of the medfly [48].

### Diet specific effects

Our results demonstrate that the beneficial effects of exposure to citrus oil depend on the dietary context. Males fed in yeast hydrolyzate and sugar, always signalled more intensively than males fed only to sugar and agree with previous studies reporting positive effects of yeast hydrolysate on signalling frequency in male medflies ([31, 32], but see also [49]). Sexual signalling in male medflies is accompanied by production and release of pheromone, which are energetically demanding [50], and thus rich food conditions enhancing both of them, could ultimately allow males to signal more frequent. Furthermore, rich food conditions (including yeast hydrolyzate) could drive sexual signalling to a plateau and as such, any differences within the tested compounds would be difficult to detect, whereas in males fed only with sugar induced effects would be more pronounced, revealing the true magnitude of each compound in enhancing male sexual signalling behaviour, as demonstrated in our study. Differential signalling pattern between males in the two contrasting diet regimes (yeast hydrolyzate and sugar versus sugar only) could be due to higher accumulation of energetic reservoirs, allowing them to proportionally signal for longer periods throughout the day and result in such shifts on the sexual signalling.

In terms of longevity we observe that absence of yeast hydrolyzate in adult diet decreases lifespan by 45.1% in male medflies (yeast hydrolyzate and sugar versus sugar only), depicting stronger effects of yeast hydrolyzate that those reported so far in male medflies [51] and comes in accordance with studies on other fruit flies [52] (but see also [49]). Interestingly, exposed males to citrus oil lived longer when they were fed only with sugar, whereas for males fed with yeast hydrolyzate and sugar a reverse pattern was observed. Our results partly come in agreement with the recent and unique work in tephritid fuit flies of Gerofotis and co-workers, that reported prolonging effects of exposure to a plant metabolite in olive fruit fly males, under poor nutritional conditions (fed only with sugar) [46]. Contrary to Gerofotis, in which exposure had no effect in males’ longevity when feeding in rich food conditions in our study males exposed to citrus oils and feeding in yeast-hydrolyzate and sugar lived significantly shorter than non-exposed. Variation between our results and in those reported in olive flies may be due to differential costs and benefits of exposure to plant volatiles that arise from the different mating system of each species. Male medflies can perform sexual signaling in the morning and at noon for several hours in each period (see Fig 4 and S1–S5 Figs) and thus sexually signalling proportionally occupies a significant period of the day, whereas the mating system in olive flies dictates one opportunity per day and thus its sexual behaviour is limited to a well-defined period at dusk (usually 1–2 hours). Male medflies (exposed and non-exposed) fed in yeast hydrolyzate and sugar perform in higher rate sexual signalling that those fed in sugar only throughout the day, indicating higher consumption of energy by yeast hydrolyzate and sugar fed males. Given that exposure to citrus oil further enhances sexual signalling in yeast hydrolyzate and sugar fed males, it is possible that any beneficial effects of exposure to citrus oil aroma in longevity cannot outweigh the cost of increased sexual signalling and thus evoke negative results, which in the case of olive fly is not possible because mating activity in this species is restricted by it’s circadian clock. Furthermore, citrus oil may activate ‘private’ mechanisms (i.e. organism-specific) of lifespan extension and not ‘public’ ones [53], whose effects are still unknown for medfly (discussed in detail by Gerofotis [46]).

### Age specific effects

The age of individuals modulated sexual signalling pattern and affected the induced effects of exposure to citrus compounds. All males (exposed and non-exposed) irrespective of the food regime clearly shifted to a more intense and continuous sexual signalling activity, in day 16^th^ of their adult age (see S2 Fig). Age is known to promote sexual maturity [26, 36] and affect mating performance and competitiveness in male medflies (28) and it could potentially also affect sexual signalling pattern [54]. However, comparisons and differences observed are between males of two age classes (8 vs 16 days old), in which most of mass-reared males are already sexual mature. Thus, we suggest another possible explanation, which is not exclusive of the above. As such, the reduced ‘relaxation’ period (between 15:00 and 18:00) in sexual calling in older males compared to young ones (see contrast between Fig 4 and S2 Fig) could be attributed to the accumulation of energetic reserves during the intermediate period (between 8^th^ and 16^th^ day of adult age), that males could subsequently channel to sexual signalling activity, in their ultimate effort to attract females and increase the changes of reproduction, especially under our experimental conditions in which males’ restriction to females could further strengthen males’ drive to attract females for mating, as time was progressing. In addition to sexual signalling pattern, sexual signalling rates of males (as a ratio of males of the two contrasting diet regimes, Fig 3) exposed to the mixture of compounds follow an increasing trend in relation to age, with a discrete peak in day 8 of their adult life. The mixture of pure compounds was capable of largely counterbalancing the negative impacts of a poor feeding environment (sugar only) on males’ sexual activity even 11 days post exposure. This indicates that the underlying mechanism affected by exposure to the mixture requires a short period of two days to induce changes in the sexual signalling and that the induced beneficial effects to sexual signalling are long lasting for at least a period of 11 days post exposure, ultimately counterbalancing the negative impacts of a poor feeding environment.

### Practical applications

The current findings may have significant practical implications for the application and effectiveness of the Sterile Insect Technique. The targets of SIT or similar methods, such as the Incompatible Insect Technique (IIT), rely on fitness characteristics (sexual competitiveness, lifespan) of field released, mass-reared, sterilized males (either through irradiation or transinfection with the endosymbiont *Wolbachia pipientis*) against feral males [1]. Females that copulate with such males lay eggs that do not develop into larvae and, therefore, the target population is suppressed. In most of these programs mass reared sterilized adults are maintained in sugar only diets before field releases [55]. Sugar fed males when exposed to the mixture of compounds performed sexual signaling in high rates, similar to those of males fed in yeast-hydrolysate and sugar. This is extremely important to any SIT program, given that the supplement of yeast-hydrolysate to adults in order to increase their sexual performance can now be achieved with an alternative cost-effective method, by replacing the provision of yeast-hydrolysate with exposure to the mixture of compounds.

Moreover, citrus oil aroma has found to increases the mating competitiveness of *C*. *capitata* males [22] and now extends its lifespan in sugar fed only males. Males under field conditions usually suffer from extrinsic mortality and rarely survive to extreme old ages (e.g. 120 days) and thus higher number of copulations during lifetime as a result of increased longevity is not expected under SIT operations. However, we cannot exclude totally this scenario, given that the prolongevity effects observed in our experiments, span through the whole spectrum of ages (see Fig 5 and Table 3) and are not depicted only in extreme old ages. Male medflies have been found to be sexually active till the age of 50–60 days old (see Papanastasiou and co-workers [28]) and thus even a small prolongation of longevity in exposed males (e.g. from 34 to 49 days) could result in a higher number of copulations. Moreover, increased longevity and enhanced mating competitiveness observed in those males indicates higher overall fitness and could also be related to other characteristics in these males related to sexual performance (e.g. ejaculate traits). Hence, large-scale, pre-release treatment of sterilized (sugar-fed) males with citrus oil might ultimately contribute to overall improvement of SIT (by minimizing the costs of diet used in holding conditions and influencing multiple quality features of produced males).

## Supporting information

S1 Table Male sexual signalling on age 8 of adult age. Repeated measures ANOVA on the effect of treatment (exposure to orange oil, limonene, mixture of 5 pure compounds and control) (first factor), food (yeast hydrolyzate and sugar and sugar only) (second factor) and time of day (repeated factor), on daily rhythms of sexual signalling on sterilized male medflies of the Vienna 8 GSS strain. Times of the day between 07:00 and 20:45 hours of adult day 8 were considered in the analysis.
(DOCX)

S2 Table Male sexual signalling on age 16 of adult age. Repeated measures ANOVA on the effect of treatment (exposure to orange oil, limonene, mixture of 5 pure compounds and control) (first factor), food (yeast hydrolyzate & sugar and sugar only) (second factor) and time of day (repeated factor) on daily rhythms, of sexual signalling on sterilized male medflies of the Vienna 8 GSS strain. Times of the day between 07:00 and 20:45 hours of adult day 16 were considered in the analysis.
(DOCX)

S1 Fig Daily pattern on male sexual signaling. Daily rhythm of sexual signalling on adult day 8 of sterilized male medflies of the Vienna 8 GSS in four different treatments (exposure to orange oil, limonene, mixture of 5 pure compounds and control) on yeast hydrolyzate & sugar (YS, left column) and sugar only (S, right column). Values on y axis indicate the mean number (±SE) of males signalling per cage. Ten cages were considered containing 10 males each.
(TIF)

S2 Fig Daily pattern on male sexual signaling. Daily rhythm of sexual signalling on adult day 16 of sterilized male medflies of the Vienna 8 GSS in four different treatments (exposure to orange oil, limonene, mixture of 5 pure compounds and control) on yeast hydrolyzate & sugar (YS, left column) and sugar only (S, right column). Values on y axis indicate the mean number (±SE) of males signalling per cage. Ten cages were considered containing 10 males each.
(TIF)

S3 Fig Daily pattern on male sexual signalling. Daily rhythm of sexual signalling on adult day 7 of sterilized male medflies of the Vienna 8 GSS in four different treatments (exposure to orange oil, limonene, mixture of 5 pure compounds and control) on yeast hydrolyzate & sugar (YS, left column) and sugar only (S, right column). Values on y axis indicate the mean number (±SE) of males signalling per cage. Ten cages were considered containing 10 males each.
(TIFF)

S4 Fig Daily pattern on male sexual signaling. Daily rhythm of sexual signalling on adult day 9 of sterilized male medflies of the Vienna 8 GSS in four different treatments (exposure to orange oil, limonene, mixture of 5 pure compounds and control) on yeast hydrolyzate & sugar (YS, left column) and sugar only (S, right column). Values on y axis indicate the mean number (±SE) of males signalling per cage. Ten cages were considered containing 10 males each.
(TIFF)

S5 Fig Daily pattern on male sexual signaling. Daily rhythm of sexual signalling on adult day 10 of sterilized male medflies of the Vienna 8 GSS in four different treatments (exposure to orange oil, limonene, mixture of 5 pure compounds and control) on yeast hydrolyzate & sugar (YS, left column) and sugar only (S, right column). Values on y axis indicate the mean number (±SE) of males signalling per cage. Ten cages were considered containing 10 males each.
(TIFF)

S6 Fig Experimental exposure device. Plastic hemisphere dome with 1-mm-diam holes with white filter paper placed in the center of the Petri dish base, in which compounds were applied.
(TIFF)

S1 File Raw data of sexual signalling. Protocol format used filled in with original observations of male sexual signaling.
(XLSX)

S2 File Raw data of male survival. Survival data of exposed and non-exposed males to citrus oil.
(XLSX)
