# Supplementary material for: Towards improving sterile insect technique: Exposure to orange oil compounds increases sexual signalling and longevity in Ceratitis capitata males of the Vienna 8 GSS
Source: PLoS One. 2017 Nov 30;12(11):e0188092. doi: 10.1371/journal.pone.0188092 (PMC5708806; doi:10.1371/journal.pone.0188092)
Supplement: S1 Table — Repeated measures ANOVA on the effect of treatment (exposure to orange oil, limonene, mixture of 5 pure compounds and control) (first factor), food (yeast hydrolyzate and sugar and sugar only) (second factor) and time of day (repeated factor), on daily rhythms of sexual signalling on sterilized male medflies of the Vienna 8 GSS strain. Times of the day between 07:00 and 20:45 hours of adult day 8 were considered in the analysis. (DOCX) [file pone.0188092.s001.docx]

**S1 Table. Effects of factors on male sexual signalling on age 8 of adult age**

| Source of variation | d.f. | MS | F | P |
| --- | --- | --- | --- | --- |
| Exposure compound | 3 | 23.10 | 14.89 | <0.001 |
| Food | 1 | 77.52 | 49.97 | <0.001 |
| Exposure compound *food | 3 | 7.60 | 4.90 | 0.004 |
| Error (between subjects) | 72 | 1.55 | - | - |
| Time of the day | 14 | 966.51 | 829.29 | <0.001 |
| Time of the day* exposure compound | 42 | 4.28 | 3.67 | <0.001 |
| Time of the day*food | 14 | 2.46 | 2.11 | 0.010 |
| Time of the day* exposure compound *food | 42 | 4.76 | 4.09 | <0.001 |
| Error (time of the day) | 1008 | 1.17 | - | - |
