# Supplementary material for: Towards improving sterile insect technique: Exposure to orange oil compounds increases sexual signalling and longevity in Ceratitis capitata males of the Vienna 8 GSS
Source: PLoS One. 2017 Nov 30;12(11):e0188092. doi: 10.1371/journal.pone.0188092 (PMC5708806; doi:10.1371/journal.pone.0188092)
Supplement: S2 Table — Repeated measures ANOVA on the effect of treatment (exposure to orange oil, limonene, mixture of 5 pure compounds and control) (first factor), food (yeast hydrolyzate & sugar and sugar only) (second factor) and time of day (repeated factor) on daily rhythms, of sexual signalling on sterilized male medflies of the Vienna 8 GSS strain. Times of the day between 07:00 and 20:45 hours of adult day 16 were considered in the analysis. (DOCX) [file pone.0188092.s002.docx]

**S2 Table. Effects of factors on male sexual signalling on age 16 of adult age**

| Source of variation | d.f. | MS | F | P |
| --- | --- | --- | --- | --- |
| Exposure compound | 3 | 18.44 | 0.97 | 0.413 |
| Food | 1 | 654.16 | 34.27 | <0.001 |
| Exposure compound*food | 3 | 17.97 | 0.94 | 0.425 |
| Error (between subjects) | 72 | 19.09 | - | - |
| Time of the day | 14 | 563.00 | 181.81 | <0.001 |
| Time of the day*exposure compound | 42 | 2.08 | 0.67 | 0.946 |
| Time of the day*food | 14 | 10.30 | 3.24 | <0.001 <0.001 |
| Time of the day*exposure compound*food | 42 | 3.11 | 1.00 | 0.467 |
| Error (time of the day) | 1008 | 3.10 | - | - |
