# Supplementary figures and images for: Towards improving sterile insect technique: Exposure to orange oil compounds increases sexual signalling and longevity in Ceratitis capitata males of the Vienna 8 GSS
Source: PLoS One. 2017 Nov 30;12(11):e0188092. doi: 10.1371/journal.pone.0188092 (PMC5708806; doi:10.1371/journal.pone.0188092)

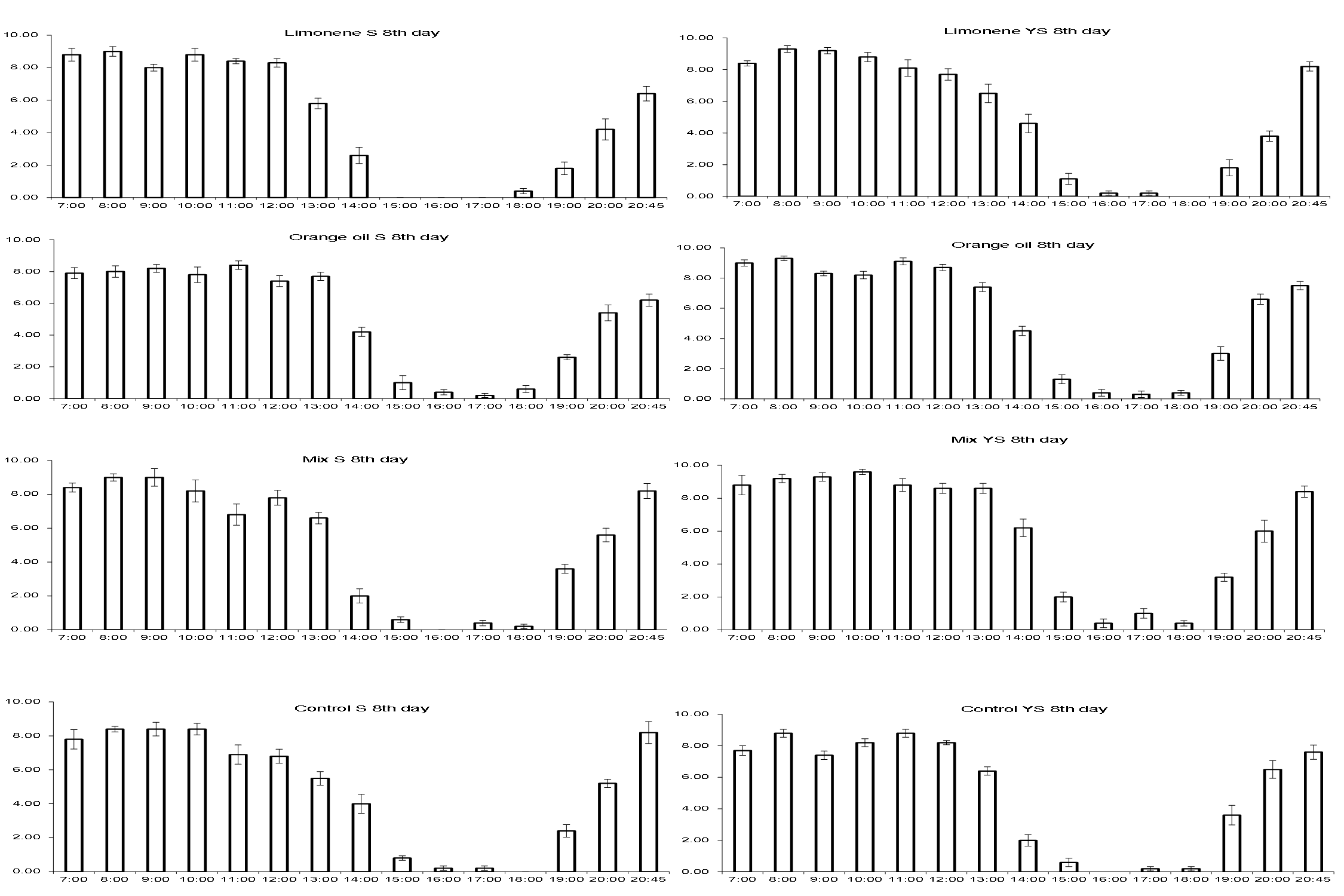

Supplement: S1 Fig — Daily rhythm of sexual signalling on adult day 8 of sterilized male medflies of the Vienna 8 GSS in four different treatments (exposure to orange oil, limonene, mixture of 5 pure compounds and control) on yeast hydrolyzate & sugar (YS, left column) and sugar only (S, right column). Values on y axis indicate the mean number (±SE) of males signalling per cage. Ten cages were considered containing 10 males each. (TIF) [file pone.0188092.s003.tif]

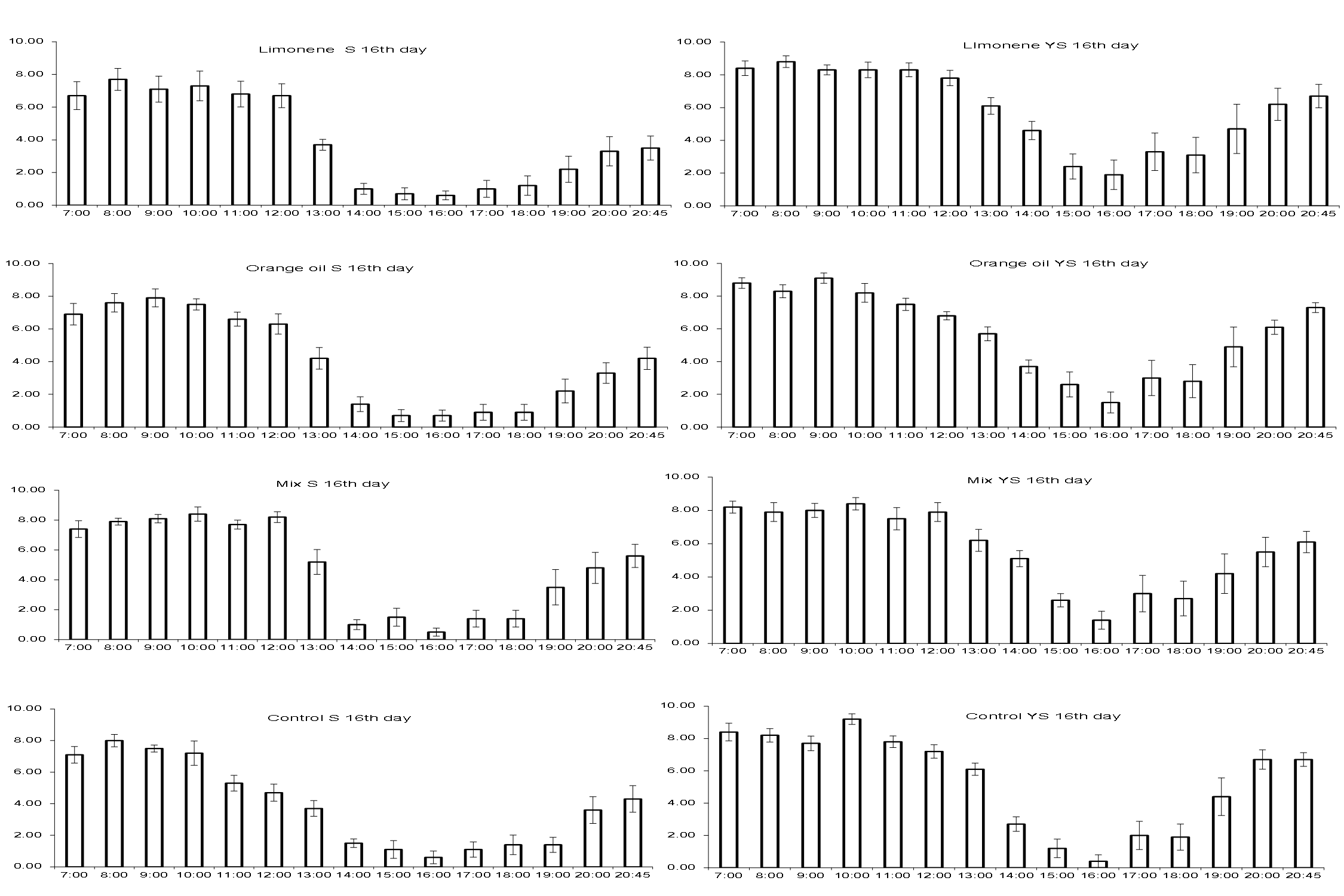

Supplement: S2 Fig — Daily rhythm of sexual signalling on adult day 16 of sterilized male medflies of the Vienna 8 GSS in four different treatments (exposure to orange oil, limonene, mixture of 5 pure compounds and control) on yeast hydrolyzate & sugar (YS, left column) and sugar only (S, right column). Values on y axis indicate the mean number (±SE) of males signalling per cage. Ten cages were considered containing 10 males each. (TIF) [file pone.0188092.s004.tif]

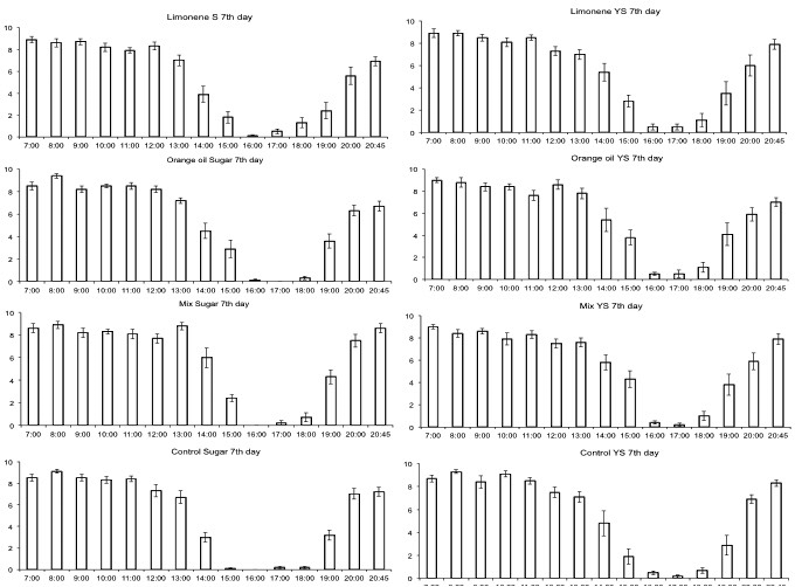

Supplement: S3 Fig — Daily rhythm of sexual signalling on adult day 7 of sterilized male medflies of the Vienna 8 GSS in four different treatments (exposure to orange oil, limonene, mixture of 5 pure compounds and control) on yeast hydrolyzate & sugar (YS, left column) and sugar only (S, right column). Values on y axis indicate the mean number (±SE) of males signalling per cage. Ten cages were considered containing 10 males each. (TIFF) [file pone.0188092.s005.tiff]

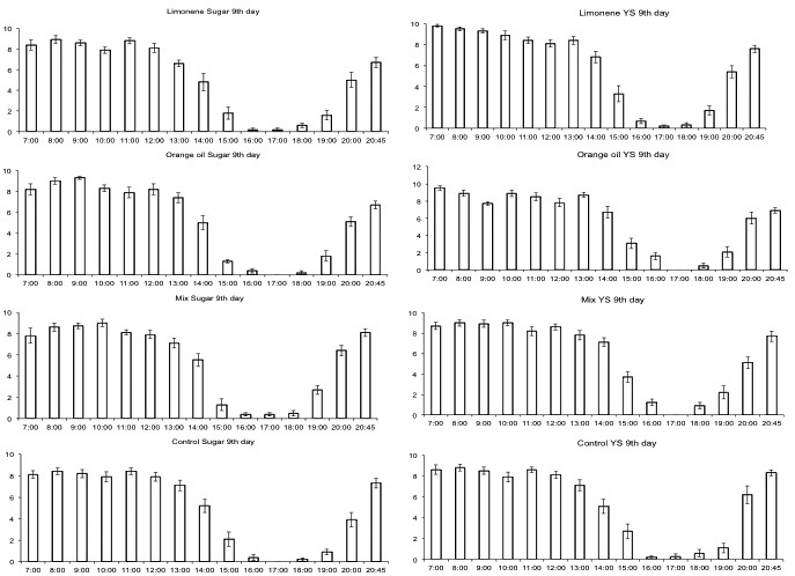

Supplement: S4 Fig — Daily rhythm of sexual signalling on adult day 9 of sterilized male medflies of the Vienna 8 GSS in four different treatments (exposure to orange oil, limonene, mixture of 5 pure compounds and control) on yeast hydrolyzate & sugar (YS, left column) and sugar only (S, right column). Values on y axis indicate the mean number (±SE) of males signalling per cage. Ten cages were considered containing 10 males each. (TIFF) [file pone.0188092.s006.tiff]

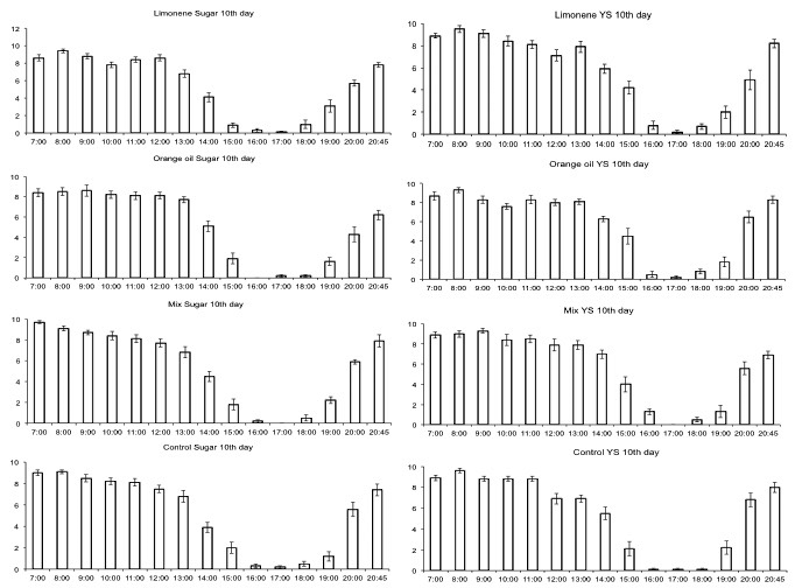

Supplement: S5 Fig — Daily rhythm of sexual signalling on adult day 10 of sterilized male medflies of the Vienna 8 GSS in four different treatments (exposure to orange oil, limonene, mixture of 5 pure compounds and control) on yeast hydrolyzate & sugar (YS, left column) and sugar only (S, right column). Values on y axis indicate the mean number (±SE) of males signalling per cage. Ten cages were considered containing 10 males each. (TIFF) [file pone.0188092.s007.tiff]

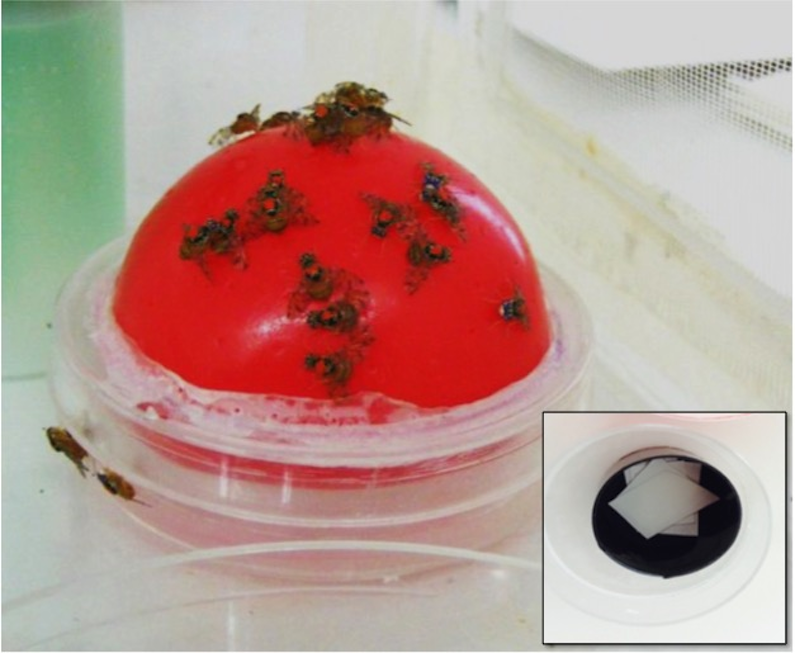

Supplement: S6 Fig — Plastic hemisphere dome with 1-mm-diam holes with white filter paper placed in the center of the Petri dish base, in which compounds were applied. (TIFF) [file pone.0188092.s008.tiff]
